# Combined Tillaux and Bimalleolar Ankle Fracture in a Pediatric Patient: A Case Report

**DOI:** 10.7759/cureus.21648

**Published:** 2022-01-26

**Authors:** Kathryn C Helmig, Timothy J Choi, Selina R Silva

**Affiliations:** 1 Orthopaedics and Rehabilitation, University of New Mexico, Albuquerque, USA

**Keywords:** open fracture reduction, bimalleolar ankle fracture, growth plate injury, ankle fracture management, tillaux fracture

## Abstract

Adolescents are at risk of unique ankle fracture patterns due to closing physes. Transitional ankle fractures, in particular, are an entity specific to adolescent patients due to the asymmetrically open distal tibia physis. Transitional ankle fractures are rarely seen in combination with bimalleolar ankle fracture patterns. This case is of interest because the combined fracture pattern and the treatment method presented have not been previously reported in the literature to our knowledge. A 15-year-old female presented with right ankle pain after a fall while roller skating. Imaging demonstrated a right Tillaux fracture with ipsilateral displaced medial malleolus fracture and minimally displaced Weber C distal fibula fracture. The Tillaux fracture and medial malleolus fractures were treated with open reduction and internal fixation with partially threaded compression screws. The lateral malleolus remained minimally displaced and did not require operative fixation. The patient healed well with no complications. Transitional injuries of the ankle in adolescents have been reported in the literature; however, combined injuries are uncommon and lack representation in the current literature base. These combined injuries are important to be aware of, as missed injuries can result in long-term pain and disability.

## Introduction

Ankle fractures in skeletally immature patients have unique characteristics regarding fracture patterns and treatment, particularly in patients with asymmetrically open physes transitioning to a mature, closed state. Transitional ankle fractures occur during progressive closure of the distal tibia physis [1]. The distal tibia physis takes 18 months on average to close completely [1-3]. Distal tibia physeal closure begins centrally, moves medially, and finishes laterally [1,2]. Distal tibia physeal closure typically occurs from age 12 to 15 in females and 14 to 18 in males [1,4].

Tillaux fractures are one type of transitional fractures which usually occur in the later stages of distal tibia physeal closure, when only the lateral portion of the physis remains open [1,3]. Tillaux fractures are typically the result of a dorsiflexion external rotation force resulting in avulsion of the anterior inferior tibiofibular ligament (AITFL) [1,2]. Bony avulsion of the AITFL results in a Salter-Harris III fracture of the anterolateral distal tibia [2,3]. Although this fracture involves the growth plate, the distal tibia physis is typically near complete closure. Therefore, physeal arrest due to Tillaux fractures does not pose a significant risk of limb shortening or deformity [1].

Bimalleolar ankle fractures are also common in pediatric patients and may or may not involve the distal tibia and fibula physes [1]. Risk of physeal arrest after bimalleolar ankle fracture depends upon skeletal maturity of the patient, mechanism of injury, initial displacement of the fracture, and residual postreduction displacement [1].

We present the case of a 15-year-old female with combined Tillaux and bimalleolar ankle fractures, a rare injury seldom discussed in the literature. There is utility in discussion of this combined fracture pattern given the uncommon injury and stepwise approach to fixation.

## Case presentation

**Figure 1:**
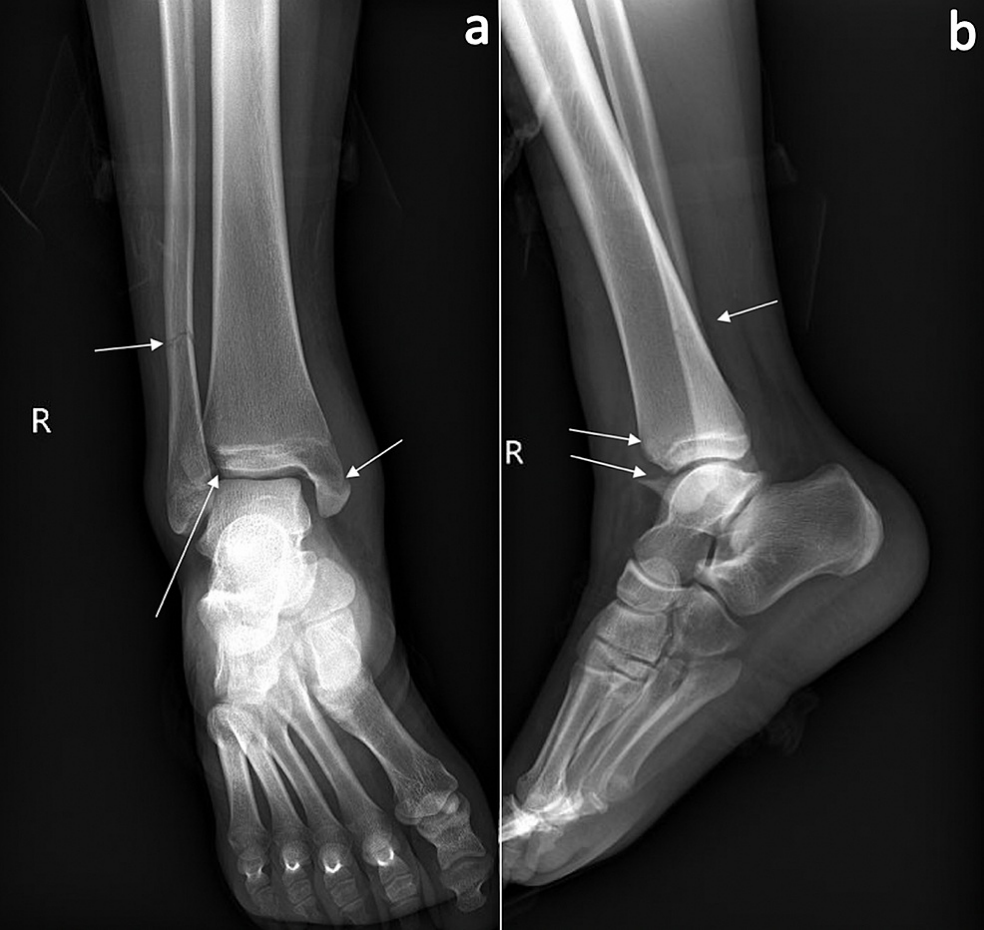
Injury radiographs Initial anteroposterior (a) and lateral (b) radiographs of the injured right (R) ankle, demonstrating displaced medial malleolus and Tillaux fractures, as well as minimally displaced distal fibula fracture (fractures indicated by white arrows).

A 15-year-old otherwise healthy female presented after an injury sustained while roller skating. Initial radiographs demonstrated combined right Tillaux and bimalleolar ankle fractures (Figures 1a, 1b).

**Figure 2:**
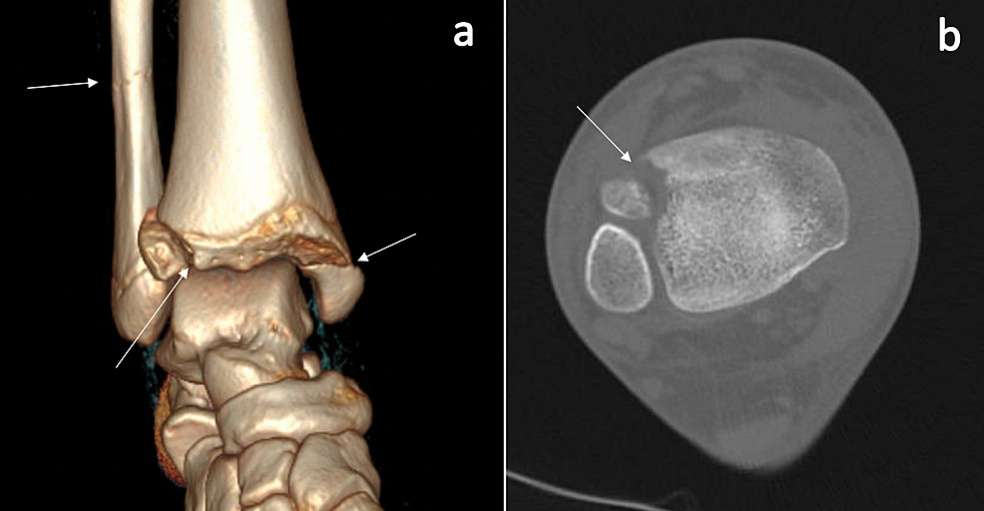
Preoperative CT scan images Preoperative three-dimensional (3D) CT scan (a) demonstrating persistent displacement of the medial malleolus and Tillaux fractures, with minimal displacement of the distal fibula fracture (fractures indicated by white arrows). Axial cut CT scan (b) demonstrating Tillaux fracture fragment displaced greater than 2 mm (indicated by white arrow).

Closed reduction was attempted, and she was placed into a short leg non-weight-bearing splint. A CT scan demonstrated greater than 2 mm of displacement of the Tillaux and medial malleolus fractures with minimal displacement of Weber C distal fibula fracture (Figures 2a, 2b).

**Figure 3:**
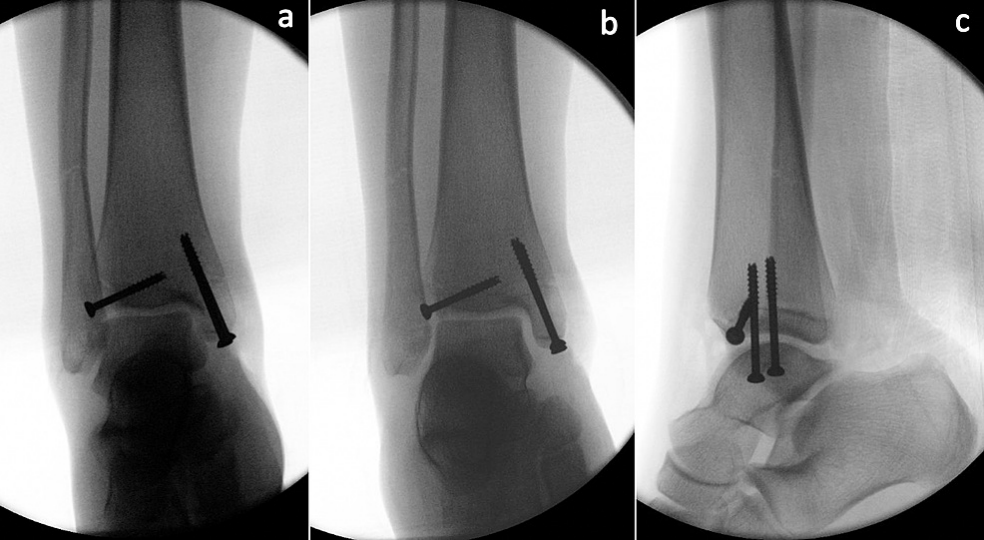
Intraoperative radiographs Intraoperative anteroposterior (a), mortise view (b), and lateral (c) fluoroscopic images demonstrating reduction of medial malleolus and Tillaux fracture fragments with appropriate placement of compression screws.

Surgical intervention was recommended due to residual displacement of the Tillaux and medial malleolus fractures. The patient was taken to the operating room for planned open reduction and internal fixation of the medial malleolus and Tillaux fractures. A stepwise approach was used, beginning with the medial malleolus. An incision just anterior to the medial malleolus was made, and the joint surface was directly visualized to ensure good reduction. The reduction was held with a two-point bone clamp, and internal fixation of the medial malleolus with two 4.0-mm cannulated, partially threaded screws was performed. A separate anterolateral incision was then made to address the Tillaux fracture. The fracture was identified, reduced, and held in place with a dental pick. A 4.0-mm cannulated, partially threaded screw was placed across the fracture with good compression noted at the fracture site. The size of the fracture fragment allowed room for only one screw. Intraoperative fluoroscopy was utilized to ensure good reduction of the medial malleolus and Tillaux fractures, appropriate screw placement, and no displacement of the fibula fracture (Figures 3a-3c).

**Figure 4:**
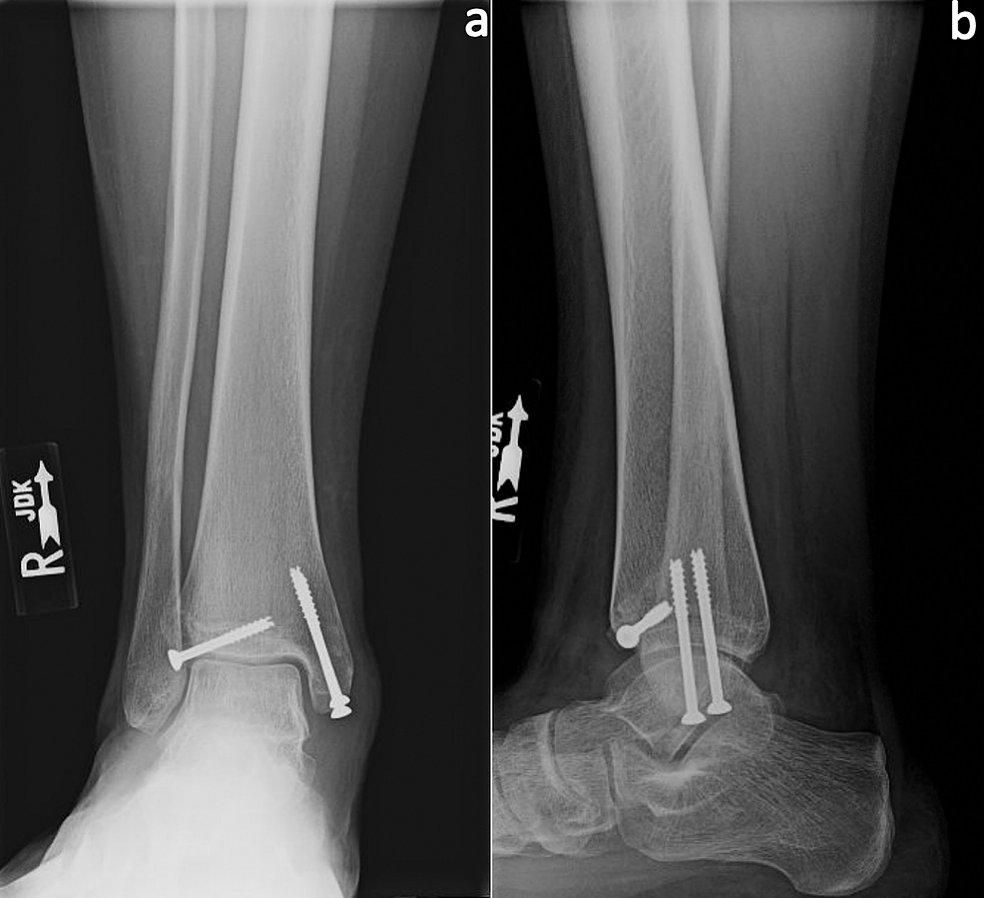
Postoperative radiographs Three-month postoperative anteroposterior (a) and lateral (b) radiographs demonstrating well-healed Tillaux and bimalleolar ankle fractures. R: right.

A short leg splint was applied, and the patient was discharged, non-weight-bearing on the operative extremity. The patient was non-weight-bearing for four weeks total, followed by weight-bearing in a walking cast for two weeks. Six weeks postoperatively, she was transitioned into an ankle brace, weight-bearing as tolerated, and began daily ankle range of motion exercises. Three months postoperatively, the patient had full painless ankle range of motion, no pain with weight-bearing, and had returned to all activities without limitations. Radiographs demonstrated well-healed distal fibula, medial malleolus, and Tillaux fractures (Figures 4a, 4b).

Given that the patient was doing well clinically and the fractures were radiographically healed at three months, further orthopedic follow-up was not indicated. On chart review, the patient established care with a pediatrician in our system, who specifically noted that she continued to have no ankle pain, swelling, or limitations 18 months postoperatively. 

## Discussion

The injury presented is an unusual combined injury; however, principles of fracture care remain the same. The primary goals in the treatment of pediatric ankle fractures are to reestablish joint congruency, physeal anatomy, and lower extremity alignment [1]. Displaced fractures (more than 2 mm) should undergo attempted closed reduction; however, repetitive attempts may cause further damage to an open physis already damaged from the injury [1,4]. If closed reduction is successful, splint or cast immobilization is applied and the patient is followed closely in clinic [1]. Regarding Tillaux fractures specifically, postreduction CT scan is indicated to evaluate for postreduction displacement as residual displacement greater than 2 mm is an indication for surgical intervention [1,4]. Adhering to principles of treatment when approaching rare combined injuries, such as in the case presented, allows a methodical and stepwise approach to the fractures. 

A review of the literature yielded four articles on Tillaux fractures combined with other ankle injuries in adolescents and adults [5-8]. Yuan et al. presented six cases of Tillaux fractures with ipsilateral medial malleolus fractures [5]. One patient underwent open reduction and internal K-wire fixation of the medial malleolus fracture, while five were treated with closed reduction and K-wire fixation [5]. All Tillaux fractures underwent open reduction and internal fixation with K-wires [5]. Five patients had ipsilateral distal fibula fractures, three of which were treated with plate and screw fixation [5]. One patient in the Yuan series underwent open reduction and internal fixation of both the medial malleolus and Tillaux fractures but did not have an ipsilateral distal fibula fracture [5]. Although one patient in the Yuan series was treated similarly to the patient presented in our case, the fixation used in the Yuan study differs from our case [5]. Partially threaded screws and K-wire fixation are both options for management of medial malleolus and Tillaux fractures. Partially threaded screws provide the advantage of compression at the fracture site, while smooth K-wires are advantageous in smaller fracture fragments at risk of comminuting with screw placement.

Multiple case reports detail combined Tillaux and Volkmann (avulsion fracture of the posterior inferior tibiofibular ligament) fractures in adolescents and adults [6-8]. This fracture pattern differs from that presented in our case but is similar in that it is a combined Tillaux fracture with additional ankle injury. The proposed mechanism of injury for combined Tillaux and Volkmann fractures is a supination external rotation force [6]. Tillaux fractures are typically associated with an external rotation force. In our case, a pronation external rotation force was most likely, based on the fracture pattern. The short oblique Weber C distal fibula fracture with transverse medial malleolus fracture indicates a pronation external rotation force on the ankle. The unusual combination fracture presented in our case may have also resulted from the patient roller skating at the time of injury and resultant atypical forces on the ankle. Mechanism of injury can be an important consideration when evaluating the patient and imaging, as the injury reported by the patient may alert the physician to evaluate the imaging for certain subtle combined injuries that may be easily overlooked.

## Conclusions

Tillaux fractures are a unique entity among pediatric patients, and the literature on this fracture pattern is limited. The literature on combined Tillaux and bimalleolar ankle fractures is even more sparse. This report demonstrates an uncommon fracture pattern and the treatment approach utilized to address the injury.

It is important for orthopedic surgeons to be aware of unusual ankle fracture patterns, particularly in adolescent patients with asymmetrically closing physes, to avoid missing a rare combined injury. Missing the diagnosis in these patients has the potential to lead to nonunion, malunion, and/or instability, resulting in long-term pain and disability. Additionally, a stepwise and methodical treatment approach based on fracture principles should be utilized when a patient with an atypical fracture pattern is encountered.
